# Wireless Indoor Localization Using Convolutional Neural Network and Gaussian Process Regression

**DOI:** 10.3390/s19112508

**Published:** 2019-05-31

**Authors:** Guolong Zhang, Ping Wang, Haibing Chen, Lan Zhang

**Affiliations:** 1 School of Automation and Electronic Engineering, University of Science and Technology Beijing, Beijing 100083, China; s20170622@xs.ustb.edu.cn (G.Z.); s20180560@xs.ustb.edu.cn (H.C.); 2 Space Star Technology Co., Ltd., Beijing 100086, China; wp6010906@163.com

**Keywords:** fingerprinting localization, received signal strength indication, k-nearest neighbor, convolutional neural network, Gaussian process regression, cumulative error distribution

## Abstract

This paper presents a localization model employing convolutional neural network (CNN) and Gaussian process regression (GPR) based on Wi-Fi received signal strength indication (RSSI) fingerprinting data. In the proposed scheme, the CNN model is trained by a training dataset. The trained model adapts to complex scenes with multipath effects or many access points (APs). More specifically, the pre-processing algorithm makes the RSSI vector which is formed by considerable RSSI values from different APs readable by the CNN algorithm. The trained CNN model improves the positioning performance by taking a series of RSSI vectors into account and extracting local features. In this design, however, the performance is to be further improved by applying the GPR algorithm to adjust the coordinates of target points and offset the over-fitting problem of CNN. After implementing the hybrid model, the model is experimented with a public database that was collected from a library of Jaume I University in Spain. The results show that the hybrid model has outperformed the model using k-nearest neighbor (KNN) by 61.8%. While the CNN model improves the performance by 45.8%, the GPR algorithm further enhances the localization accuracy. In addition, the paper has also experimented with the three kernel functions, all of which have been demonstrated to have positive effects on GPR.

## 1. Introduction

With the rapid growth of the Internet of Things market, indoor localization has long been a question of great interest in a wide range of fields. There is an urgent need to address the precise indoor localization problems caused by location-based services. Location-based services typically include indoor navigation, shop finding, targeted advertising, transportation, users flow analysis and other industrial fields [1,2,3]. For localization in an outdoor environment, a Global Navigation Satellite System (GNSS) is an ideal method that meets people’s performance requirements. However, the signals from GNSS have proven to be unreliable in an indoor environment. Therefore, we urgently need positioning methods that can perform well in indoor environments [4,5].

Various methods applied to provide a localization service have different accuracies in different kinds of indoor environments. Existing research recognizes the critical role played by received signal strength indication (RSSI) based localization methods. Many RSSI based systems require periodic calibrations and information fusion which decreases their performance [6]. The RSSI-based localization method, whether ranging-based or not, is still the mainstream research method. For example, a RSSI ranging-based algorithm utilizing the radio and search and the least squares algorithm can be used for partial discharge source localization [7]. Besides, Wi-Fi fingerprinting localization is one of the methods based on RSSI in wireless sensor networks (WSNs) [8,9]. Compared to other indoor localization methods, Wi-Fi fingerprinting localization technology has some advantages including low hardware requirement and wide scope of application. At the same time, the technology needs to cooperate with more advanced algorithms to ensure higher positioning precision.

Fingerprinting-based localization algorithms are usually either deterministic or probabilistic algorithms. As for deterministic algorithms, previous studies have successfully manifested the crucial role played by the k-nearest neighbor (KNN) algorithm and its variants including the weighted KNN (WKNN) algorithm in a fingerprinting-based localization area [10]. In [11], Shin et al. found that the enhanced WKNN reduces the error compared to KNN by adjusting the number of considered neighbors. In [12], Fang et al. proposed an optimal WKNN algorithm composed of an adaptive Kalman filter and a memetic algorithm. These advanced algorithms based on WKNN have improved the localization performance compared to the KNN algorithm. Besides, previous studies have revealed that support vector machines (SVM) are also an effective means to solve Wi-Fi fingerprinting localization. Abdou et al. [13] presented the combining of SVM and cluster as a regression localization algorithm. The algorithm first analyzed the data in the fingerprinting database by clustering and then employed SVM to establish the intrinsic relationship between locations and fingerprints. However, when the dimension of the RSSI vectors is large, the SVM algorithm will significantly increase in time complexity. As for probabilistic algorithms, they commonly build a probability distribution model on the RSSI values in the fingerprinting database firstly and then apply probabilistic algorithms to estimate the locations of test points (TPs) [14,15,16]. In [17], Piotr et al. focused on the Kullback-Leibler divergence metric that compared multivariate RSSI distributions to provide accurate location estimates. A Gaussian process (GP) was presented as a likelihood model for RSSI values [18]. Many probabilistic algorithms, including Gaussian processes, give confidence intervals for predicted values, however do not achieve ideal localization accuracy. Therefore, the research of KNN-based localization algorithms is more common.

Prior studies have not been able to convincingly manifest that a proposed algorithm will achieve an ideal performance in real-world scenarios. With a focus on reflecting the effect of the algorithm in a complex environment, we chose a fingerprinting database collected in the library of Jaume I University as the data source of this paper. Additionally, high-dimensional RSSI vector in this database can better verify the performance of the algorithm.

In this paper, we propose a wireless positioning hybrid model using both convolutional neural networks (CNN) and Gaussian process regression (GPR). Recently, CNN has been applied to related research in indoor localization. Several studies, for instance [19] and [20], were carried out on investigating the effectiveness of using CNN for visual localization. CNN is commonly employed for image recognition because they could be thought of automatic feature extractors of images by using adjacent pixel information to effectively subsample the image first by convolution and then adopting a prediction layer at the end [21]. Similarly, we set the fingerprinting dataset as several “image”. A number of RSSI vectors of a location at different times were chosen to form an “image”. Each RSSI vector was treated as a multi-channel pixel in the “image”. Finally, each RP in the fingerprinting dataset got its own “image” which will be utilized in the further algorithm. In the first phase of the model, we used CNN to extract reliable features of “images” and then built internal representation between “images” and locations of reference points (RPs) based on the Pytorch computational framework. In the second phase, we evaluated the estimation error of the trained CNN model by validation points (VPs), then trained the GPR algorithm by the error with their respective fingerprints of VPs. Finally, we employed a trained GPR model to correctly estimate coordinates of CNN in order to improve the location accuracy. GPR is a widely accepted algorithm for solving nonlinear regression problems for its probabilistic benefits and ability to put high-dimensional data within reach. Combining the advantages of CNN and GPR algorithms, our proposed hybrid model effectively mitigates multipath effects, other noises and human body interference on Wi-Fi signals, improving positioning accuracy.

The main contribution of this paper is as follows:

- A fingerprinting-based localization hybrid model using both the CNN and GPR algorithm.
- Performance evaluation of the proposed model and its comparison with the KNN algorithm.
- The comparison of positioning performance between the hybrid model based on different kernel functions.

The remaining part of the paper proceeds as follows: Section 2 begins by introducing the Wi-Fi fingerprinting localization technology analysis and then analyses the data gathered, focusing on signals pre-processing. Section 3 is concerned with the proposed model including the structure and other details about CNN and GPR models. In particular, Section 3.5 contextualizes the GPR model by fingerprinting-based localization and highlights the key concept. Section 4 presents the experimental results and discusses the significant findings. The final section gives a summary and critique of the findings.

## 2. Wi-Fi Fingerprinting Localization

### 2.1. Technology Analysis

In recent years, there has been an increasing interest in algorithms based on fingerprinting localization technology [22]. The reason for the above phenomenon is related to the characteristics of fingerprinting localization. On the one hand, it has the advantages of being low-cost and having wide applications in indoor scenarios. A general indoor scenario where enough intense Wi-Fi networks are deployed without any other hardware could be an ideal place to apply the technology. On the other hand, the disadvantages of the technology include high requirements for data support and algorithm quality to ensure accuracy. Admittedly, there are two types of solutions including increasing collecting density of RPs and improving the algorithm to find better matching function between locations of RPs and their respective RSSI vectors [23]. From the aspect of practice, the former solution will increase costs which come from setting more access points (APs) in advance, taking more time to collect the fingerprinting data and processing more complex information. Consequently, paying more attention to an approved algorithm is a more economical choice. In addition, a specific fingerprinting database is required to solve the localization problem of a particular scene. Once the localization environment changes, the fingerprinting database needs to be re-acquired.

Other than that, there are some influence factors of Wi-Fi fingerprinting localization technology.

**Figure 1:**
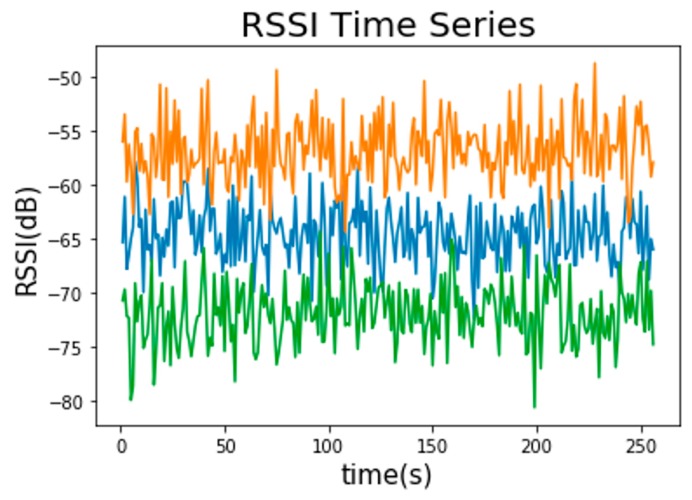
The variation of received signal strength indication (RSSI) values from three access points (APs).

- The position and movement of the human body in an indoor environment can trigger signal fluctuations [24]. This is even more crucial in public areas where there is a large flow of people [25]. As is exhibited in Figure 1, the RSSI values from three APs fluctuate over a period of time. It manifests that the RSSI value is not very stable.

As is exhibited in Figure 1, the RSSI values from three APs fluctuate over a period of time. It manifests that the RSSI value is not very stable. The RSSI data in Figure 1 is derived from a simple experiment. Three APs are placed in the experimental space. The RSSI data was collected by a trained person that stood at a fixed position, holding the mobile device and receiving signals from three APs over a period of time. The distance between the three APs and the mobile device is different, resulting in inconsistent strengths of the three groups of signals in Figure 1 where the orange signal comes from the nearest AP while the green signal comes from the farthest AP.

- Multipath effect is the major factor affecting indoor localization accuracy because of signals received from different paths [26].
- There is a difference in the orientation of the collector or user when collecting signals [25].

All of these factors affect the fingerprinting database and ultimately have impacts on positioning accuracy.

### 2.2. Wi-Fi Fingerprinting Data Collection

**Figure 2:**
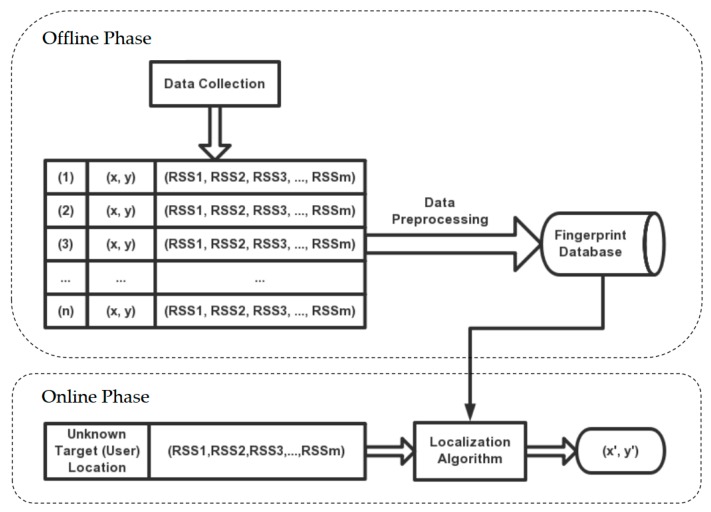
Two phases of fingerprinting localization.

Wi-Fi fingerprinting localization is also known as scene analysis. Commonly, scene analysis consists of two phases, as presented in Figure 2, including the offline training phase and online localization phase. In the offline training phase, WSNs sensor modules are placed in an exactly indoor environment on fixed locations (access points). Then, a trained person stands at predefined locations (reference points), holding the mobile device and collecting the RSSI values from APs. The collection proceeds through a list of predefined locations. Finally, these collected RSSI values and their corresponding coordinates compose a fingerprinting database or are called a radio map for estimating the locations of target points, which include users with smartphone or robots in the WSNs environment [27]. During the online localization phase, researchers collect the RSSI values of users and match them to the fingerprinting data in the radio map by a localization algorithm to estimate the user’s location. In the online localization phase, testing samples consist of the RSSI values collected by the user at unknown target locations. For the estimation of the target locations, a localization algorithm is used in matching the testing samples to the fingerprinting data in the radio map [28].

Besides, most of the studies are evaluated in a specific environment or on a private dataset which hampers the reproducibility of the experimental results and has a negative impact on the comparison between different algorithms [12,27,28]. For the purpose of foster comparison and development in indoor localization research, some databases have been made available to the public. However, there are two major problems with most of the current databases. Firstly, it is difficult to maintain the database for a long time. Secondly, it is difficult to apply the database to new algorithms.

In order to handle the mentioned problems, a long-term Wi-Fi fingerprinting database for the library of Jaume I University was selected for performance evaluation in this paper [29]. The origin version of this database was founded on 24 November, 2017 and has been updated twice. The latest version 2.1 was uploaded on 6 November, 2018 on Zenodo. The data collection spanned over 15 months on version 1.0 and 25 months on version 2.1. Experiments in the paper are based on version 2.1. 

**Figure 3:**
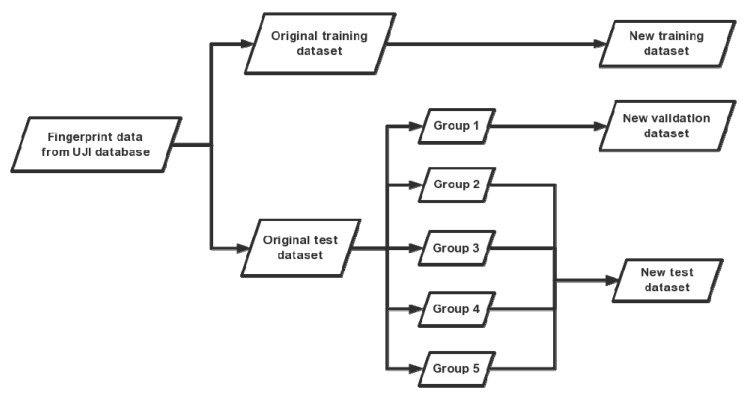
Dataset reorganization.

Specifically, collecting data of each month from the database involved a training dataset and five groups of test datasets. According to Figure 3, the original training and test dataset are divided into three datasets including new training dataset, new validation dataset and new test dataset. Firstly, the original training dataset was considered as the new training dataset which would be adopted in training a CNN model. Then, different from the usage in [19], the original test dataset was divided into two parts including new validation dataset that consisted of the first group of original test datasets and a new test dataset that consisted of other groups of original test datasets. The validation dataset would be adopted in training a GPR model. The datasets that appear below refer to new datasets without an additional explanation.

### 2.3. Signals Pre-Processing

For the purpose of training CNN, the data processing focused on transforming the data into a desired form which would be successfully understood and utilized.

In order to improve training efficiency, the following steps were taken prior to transformation. Firstly, the RSSI values were set to zero if they were below −105 dBm and, secondly, all of the remaining signals were normalized. As for the first step, signals value less than this threshold usually means that the communication distance is too far to provide valuable information for localization. Meanwhile, the Wi-Fi-based RSSI values are susceptible to noise interference so that signals below the threshold would be unreadable. As for the second step, min–max normalization was applied in the remaining signals as

(1) $${\tilde{x}}_{i}=\frac{x_{i}-x_{\min}}{x_{\max}-x_{\min}}.$$

Furthermore, the data collected during 25 months were reorganized according to the dataset that they belong to instead of the month they were collected. As a result, the training dataset contained coordinates of 24 RPs and 300 RSSI vectors, respectively. The validation dataset contained coordinates of 24 VPs and 300 RSSI vectors, respectively. The test dataset contained coordinates of 82 TPs and 300 RSSI vectors, respectively. Therefore, each point and its corresponding numbers of RSSI vectors constitute the Wi-Fi fingerprinting database adopted in this study.

After the above pre-processing steps, normalized signal vectors were organized into a desired form. The first 256 RSSI vectors of each point including all of RPs, VPs and TPs from each dataset were shaped as a 16 × 16 matrix. Specifically, the selected 620-dimensional vectors were sequentially arranged as a matrix with a height of 16, a width of 16 and a depth of 620. Moreover, the form of the fingerprinting database were changed as coordinates of 24 RPs and 24 corresponding matrixes in the training dataset, coordinates of 24 VPs and 24 corresponding matrixes in the validation dataset and coordinates of 82 TPs and 82 corresponding matrixes in the test dataset.

In a typical CNN, the training data is usually a single-channel grayscale image or a three-channel color image [30]. Similarly, the training data in this study can be considered as multi-channel “images”. Different from the real images, the number of channels in the “images” in this paper reaches 620.

## 3. Proposed Model

### 3.1. Model Structure

**Figure 4:**
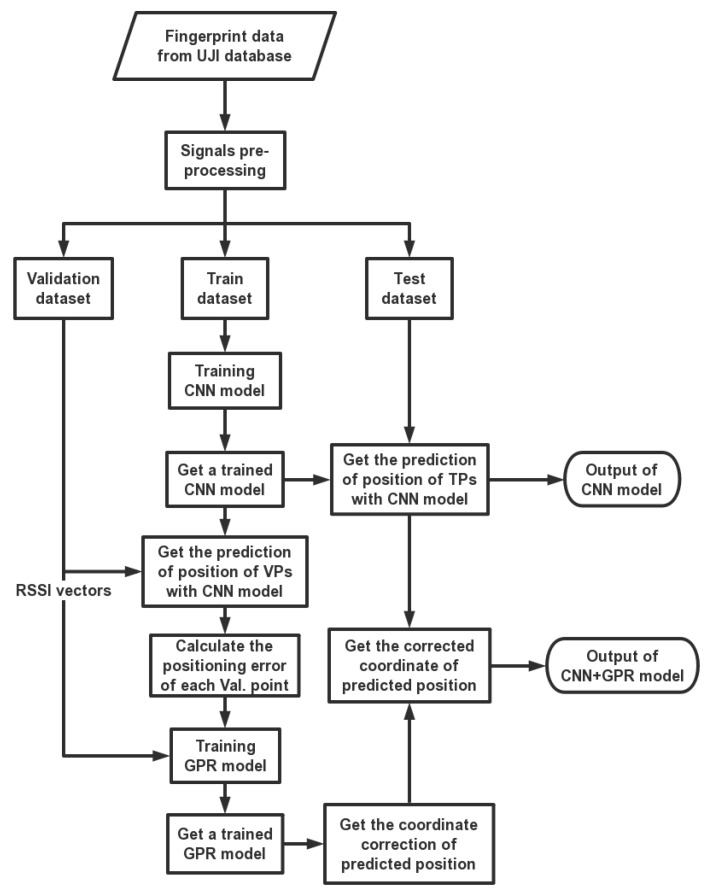
The structure of the proposed model.

The structure of the model is illustrated in Figure 4. The pre-processing database is divided into three parts: training dataset, validation dataset and test dataset. They are adopted in training and testing CNN or GPR in different phases.

In the first phase of the model, the training dataset was adopted in CNN training, then we got a trained CNN model. In the second phase, we first employed the validation dataset to evaluate the accuracy of the CNN model’s position prediction for VPs and to calculate the positioning error. Then, we trained the GPR model by the RSSI vectors from the validation dataset and the positioning error. Finally, we utilized the trained GPR model to correct the coordinates of the CNN algorithm’s position prediction of TPs for the purpose of improving the localization accuracy.

Many researchers have utilized CNN for image classification. A unified CNN-RNN framework is utilized for multi-label image classification [31]. A hybrid approach combines deep CNN and unsupervised aggregators was proposed in image classification for reducing learning cost [32]. However, few researchers employ the algorithm for indoor localization. In fact, CNN plays a unique role in studying indoor localization [33]. Existing CNN-based localization algorithms are not applicable to general Wi-Fi fingerprinting data. The model proposed in this paper should convert the general Wi-Fi fingerprinting data into the required form while ensuring performance after positioning. Furthermore, notwithstanding that the CNN model exhibits ideal localization performance, the localization model integrating CNN and GPR presents better positioning performance than using the CNN algorithm alone.

### 3.2. Convolutional Neural Network Architecture and Training

**Figure 5:**
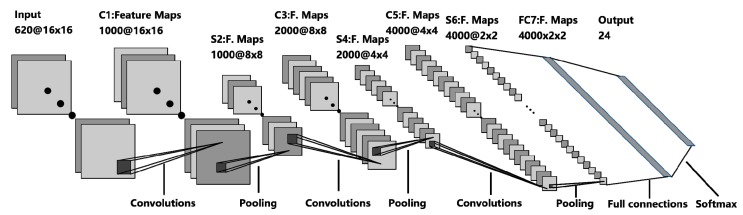
Proposed convolutional neural network (CNN) model architecture.

The proposed CNN model was employed as a probabilistic estimator. The network was organized in a layered architecture as summarized in Figure 5. It has seven layers including three convolutional layers, three pooling layers and a fully-connected layer. The input layer is the 16 × 16 × 620 “images” from the training dataset. For the first convolutional layer, 16 × 16 × 1000 feature maps are created by 1000 3 × 3 filters with a rectified linear unit (ReLU) activation function. The padding and stride are both set to one. The convolutional layer is followed by the functional layers. Spatial reduction layer is one of the functional layers. Maximum pooling spatial reduction functional layer is selected to progressively reduce the spatial size, resulting in the reduction of the computational complexity of the network and prevention of overfitting to some extent. Moreover, the pooling layer separately shrinks each of the input depth slices in space. The most general form, 2 × 2 (width and height) pooling window with a stride of two, is set to perform subsampling which selects the max over four number and then shifts for two pixels. After that, the volume of each “image” changes to 8 × 8 × 1000. The depth dimension of each “image” stays constant during the pooling process.

For the second convolutional layer, 2000 3 × 3 filters with ReLU activation function are applied to create 8 × 8 × 2000 feature maps. Similarly, another maximum pooling layer is adopted in the meantime which resizes the representations as 4 × 4 × 2000. For the third convolutional layer, 4 × 4 × 4000 feature maps are built by 4000 3 × 3 filters with ReLU activation function. Then, maximum pooling layer with 2 × 2 pooling window reduces the volume to 2 × 2 × 4000.

After a series of convolutional and spatial reduction layers, a fully connected layer occurs. A combination of convolutional layers with spatial reduction layers work as an automatic input preprocessing unit that replaces the traditional complex feature extraction procedures. They serve as an automatic feature extraction layer providing features to the following fully connected neural layer. A fully connected layer has all the neurons connected to all outputs of the last convolutional layer. Then, a readout layer calculates the corresponding output.

Finally, a layer with softmax activation was employed to yield probabilities for each RP. For a classifier, the maximum probability in this vector was selected to determine the classification of the image. As can be seen in Figure 5, the architecture of the CNN algorithm is shown above. In this paper, the probabilities for each RP are considered as the weights for each reference coordinates. Therefore, the estimated coordinate of the target point is the weighted sum of the reference coordinates.

Dropout was set to 0.5 for the convolutional layers and for the fully connected layer in order to prevent over-fitting in the model. The loss function was set to minimize the categorical cross-entropy using Adam optimizer. The training was performed with a batch size of 50 and 30 epochs.

The CNN model was built and evaluated in Python 3.7 using Pytorch package.

### 3.3. Error Correction Model Using GPR

Generally speaking, validation dataset is usually adopted as a fake test dataset to adjust the hyper-parameters. Specifically, it was employed to train the GPR model in this paper. In an effort to further prevent CNN from over-fitting the training data, the trained GPR model was applied to further improve the localization accuracy. More specifically, some related steps will be shown as follows.

Prior to training a GPR model, the validation data was applied to evaluate the performance of the trained CNN model. The localization error of the CNN model was obtained by calculating the coordinate difference between the actual location and the predicted location. The training dataset format required for the GPR model is different from the existing dataset format. Hence, a new training dataset for GPR was built:

- Input data: 24 sets of 620-dimensional vectors from the validation dataset. Each set contains 16 × 16 signal vectors and 24 sets contain a total of 6144 signal vectors.
- Output data: 24 sets of two-dimensional vectors from the localization errors between the predicted coordinates from the trained CNN and their real coordinates. Since each set of input data corresponds to the same VP, the input data (signal vector) of the same set corresponds to the same output data (error vector). Therefore, the output data includes a total of 6144 error vectors.

Following that, we proposed to utilize the GPR algorithm to establish the relationship between the input data and the output data from the GPR training dataset. The input data is several high latitude vectors which results in a complex nonlinear relationship between the input data and the output data. GPR has the natural advantage of dealing with complex models. It is a non-parametric algorithm which means that it can be expressed as any functional form, which makes it useful in dealing with any complex models. Since training data is processed into a form that is suitable for CNN, the training set becomes smaller. However, most machine learning algorithms need to rely on big data to improve the accuracy of their predictions. Fortunately, the GPR algorithm is a good choice if the application scenario is a highly nonlinear model and has a small training set. Therefore, we utilized the non-parametric Gaussian process regression algorithm to build the error correction model.

### 3.4. GPR in Function-Space View

Generally, a Gaussian process is usually specified by its mean function and covariance function [34]. We defined the mean function μ(x) and the covariance function $k(x,x^{\prime })$ of a real process f(x) as,

(2) $$\left\{ \begin{array}{l} \mu (x)=E[(x)]\\ k(x,x^{\prime })=\mathrm{cov}(f(x),f(x^{\prime })) \end{array}\right.,$$

which can be written as

(3) $$f(x)\sim GP(\mu (x),k(x,x^{\prime }))$$

A GP is defined as a collection of random variables. Usually, *x*, the index set of the random variables is time, which represents that the study object is a set of sequences about time. However, the index set *x* in this paper is RSSI, $x\in {\mathbb{R}}^{d},d=620$, rather than time.

We consider our model in such a simple form

(4) y=f(x)+ω.

where $f(x)\sim GP(\mu (x),k(x,x^{\prime }))$ and $\omega \sim N(0,\sigma_{n}^{2})$. ω is additive Gaussian noise with zero mean and variance $\sigma_{n}^{2}$. The acquisition of $\sigma_{n}^{2}$ will be demonstrated in Section 3.6. We have n = 6144 pairs of training data $D={\left\{\left(x_{i},y_{i}\right)\right\}}_{i=1}^{n}$, where each input data $x_{i}\in {\mathbb{R}}^{d}$ is a signal vector and each output data $y_{i}\in {\mathbb{R}}^{2}$ is a localization error vector. For notational convenience, we aggregate the n input vectors $x_{i}$ into a d×n matrix X and output vectors $y_{i}$ into y. The GP defines posterior distributions over function from input data to output data which indicates that $\left[f(x_{1}),f(x_{2}),f(x_{3}),\cdots ,f(x_{n})\right]$ obeys a multivariate Gaussian distribution.

(5) $$\left\{ \begin{array}{l} {\left[f(x_{1}),f(x_{2}),f(x_{3}),\cdots ,f(x_{n})\right]}^{T}\sim N(M,K_{ij})\\ M={\left[\left(\mu (x_{1}),\mu (x_{2}),\mu (x_{3}),\cdots ,\mu (x_{n})\right)\right]}^{T}\\ K_{ij}=k(x_{i},x_{j}) \end{array}\right.$$

where *M* is the mean vector and *K* is the kernel function $k(x_{i},x_{j})$ of $i^{th}$ and $j^{th}$ element which is represented by an n×n covariance matrix of inputs X. These distributions are represented non-parametrically. A key idea underlying GP is the requirement that the function values at different points are correlated. We aim to predict the function value (denoted as $f_{*}$) of testing RSSI vectors (denoted as $X_{*}=\left[x_{*1},x_{*2},\cdots ,x_{*m}\right]$), conditioned on training data D. In our GPR model, training inputs and testing vectors have the same dimension, which means m = n. The joint distribution of predictive object $f_{*}$ and the noise-free observations in the training set can be obtained as

(6) $$\left[\begin{array}{c} y\\ f_{*} \end{array}\right]\sim N\left(\left[\begin{array}{c} M(X)\\ M(X_{*}) \end{array}\right],\left[\begin{array}{cc} K(X,X)+\sigma_{n}^{2}I & K{\left(X_{*},X\right)}^{T}\\ K(X_{*},X) & K(X_{*},X_{*}) \end{array}\right]\right)$$

where $M(X)={\left[\left(\mu (x_{1}),\mu (x_{2}),\cdots ,\mu (x_{n})\right)\right]}^{T}$, $M(X_{*})={\left[\left(\mu (x_{*1}),\mu (x_{*2}),\cdots ,\mu (x_{*n})\right)\right]}^{T}$, $K(X,X)=K_{ij}$, $K_{ij}(X_{*},X)=k(x_{*i},x_{j})$ is the kernel function of $i^{th}$ and $j^{th}$ element, respectively, which is represented by an m×n covariance matrix between testing vectors $x_{*i}$ and training input while $K_{ij}(X_{*},X_{*})=k(x_{*i},x_{*j})$ is represented by an m×m covariance matrix of testing vectors. According to the theorem of marginalization and conditional distribution, the predictive distribution of target can be obtained as

(7) $$\left\{ \begin{array}{l} p(y_{*}|X,y,X_{*})=N(\hat{M},\hat{\Sigma })\\ \hat{M}=K{\left(X_{*},X\right)}^{T}{\left(K(X,X)+\sigma_{n}^{2}I\right)}^{-1}(y-M(X))+M(X_{*})\\ \hat{\Sigma }=K(X_{*},X_{*})-K{\left(X_{*},X\right)}^{T}{\left(K(X,X)+\sigma_{n}^{2}I\right)}^{-1}K(X_{*},X) \end{array}\right.$$

Taking noisy observations into account, the predictive distribution will be obtained from the training set and testing RSSI vectors as

(8) $$p(y_{*}|X,y,X_{*})=N(\hat{\mu },\hat{\Sigma }+\sigma_{n}^{2}I).$$

In addition, the predictive expression of mean function and variance function will be more concise if we adjust the mean function of the training set to zero.

(9) $$\left\{ \begin{array}{l} \hat{M}=K{\left(X_{*},X\right)}^{T}{\left(K(X,X)+\sigma_{n}^{2}I\right)}^{-1}y\\ \hat{\Sigma }=K(X_{*},X_{*})-K{\left(X_{*},X\right)}^{T}{\left(K(X,X)+\sigma_{n}^{2}I\right)}^{-1}K(X_{*},X) \end{array}\right..$$

The predictive distribution in (9) summarizes the key advantages of GP for the localization error likelihood model. The GP posterior is estimated from acquired signal vectors to localization error vector, assuming independence between different signal vector separately. During localization, the likelihood of observing localization error vector can be computed at any signal vector using (9).

### 3.5. Kernel Function

The concept of kernel is central to GPR for predicting the testing targets. It is kernel that encodes presumptions about the function that we hope to know. As for the presumptions, similarity between input data x is the major problem due to closer input data being possible to have similar output data. Therefore, it is feasible to utilize similarity between training points and TPs as a guidance about the prediction of targets. We have selected three kernels that may be suitable for the RSSI vectors: Squared Exponential (SE), Periodic (PER) and Matern [35].

#### 3.5.1. Squared Exponential

The squared exponential is one of the most well-known kernel functions used for GPR, as shown below

(10) $$k_{SE}(x,x^{\prime })=s_{f}^{2}\exp(-\frac{\left\|x-x^{\prime }\right\|}{2l^{2}}).$$

where $s_{f}^{2}$ is the signal variance and is also considered as an output-scale amplitude and parameter l is the input length-scale that determines the strength of the correlation between inputs. The meaning of these two parameters of the latter two kernel functions is the same as this kernel function. How these parameters are derived from the training data D will be explained in Section 3.6. Before training the GPR model, the variance is set to 1.0 and the length-scale is set to 0.2. The most striking feature of this kernel function is that it is very smooth. One of the reasons is that it is infinitely differentiable. In other words, the GP with SE kernel function can perform a mean-square derivation at any order.

#### 3.5.2. Periodic

The periodic kernel is employed to model functions that exhibit a periodic pattern.

(11) $$k_{PER}(x,x^{\prime })=k_{SE}(\varpi (x),\varpi (x^{\prime }))=s_{f}^{2}\exp\left(-\frac{2\sin^{2}\left(\pi \frac{\left(x-x^{\prime }\right)}{p}\right)}{l^{2}}\right).$$

where $\varpi (x)={\left[\sin(\pi x/p),\cos(\pi x/p)\right]}^{T}$ and p is the period. In the study, the variance is set to 1.0, the length-scale is set to 0.2 and the period is set to 1.0. Signal vectors may appear periodic locally, so periodic kernel function may be applicable to this model.

#### 3.5.3. Matern Class

The Matern class of kernel function is given by

(12) $$k_{Matern}(x,x^{\prime })=s_{f}^{2}\frac{2^{1-v}}{\Gamma (v)}{\left(\frac{\sqrt{2v}(x,x^{\prime })}{l}\right)}^{v}K_{v}\left(\frac{\sqrt{2v}(x,x^{\prime })}{l}\right)$$

with positive parameters *v* and *l*, where $K_{v}$ is a modified Bessel function. It is possible that the most interesting cases for machine learning are v=3/2 and v=5/2, for which

(13) $$\left\{ \begin{array}{l} K_{3/2}(x,x^{\prime })=\left(1+\frac{\sqrt{3}(x,x^{\prime })}{l}\right)\exp\left(-\frac{\sqrt{3}(x,x^{\prime })}{l}\right)\\ K_{5/2}(x,x^{\prime })=\left(1+\frac{\sqrt{5}(x,x^{\prime })}{l}+\frac{5{\left(x,x^{\prime }\right)}^{2}}{3l^{2}}\right)\exp\left(-\frac{\sqrt{5}(x,x^{\prime })}{l}\right) \end{array}\right.$$

Regarding the setting of the parameter v, it is usually set between v=1/2 and v=7/2. If v≤1/2, the process will be too rough to get an ideal model. When there is no clear prior knowledge about the existence of higher order derivatives, the limited noisy training examples will be the main problem if we set v≥7/2. After taking the above issues into account and comparing the commonly used values v=3/2 and v=5/2, we set v=3/2. At the same time, the variance was set to 1.0 and the length-scale is set to 0.2.

### 3.6. Hyperparameter Estimation

We can set $\theta =\left(\sigma_{n}^{2},s_{f}^{2},l\right)$ as the hyperparameters. In periodic kernel, $\theta =\left(\sigma_{n}^{2},s_{f}^{2},l,p\right)$. The log likelihood of the observations is given by

(14) $$-\log p(y|X,\theta )=\frac{1}{2}(y-M(X){)}^{T}{\left(K+\sigma_{n}^{2}I\right)}^{-1}(y-M(X))+\frac{1}{2}\log\left|K+\sigma_{n}^{2}I\right|+\frac{n}{2}\log2\pi ,$$

The process of obtaining the hyperparameter can be transformed into a process of obtaining the minimum value of (14). Such a process can be implemented by utilizing conjugate gradient descent. Specifically, we can compute the partial derivatives of (14).

(15) $$\frac{\partial }{\partial \theta_{j}}\log p\left(y|X,\theta \right)=\frac{1}{2}tr\left(\left(K^{-1}y\right){\left(K^{-1}y\right)}^{T}\frac{\partial K}{\partial \theta_{j}}\right)$$

Next, the corresponding partial derivatives are calculated according to different kernel functions. For example, the partial derivatives of each element for squared exponential kernel function is as follows

(16) $$\{\begin{array}{c} \frac{\partial K}{\partial \sigma_{n}^{2}}=2\sigma_{n}\delta \\ \frac{\partial K}{\partial l}=s_{f}^{2}\exp\left(-\frac{1}{2}{\left(\frac{x-x^{\prime }}{l}\right)}^{2}\right)\frac{{\left(x-x^{\prime }\right)}^{2}}{l^{3}}\\ \frac{\partial K}{\partial s_{f}^{2}}=2s_{f}^{2}\exp\left(-\frac{1}{2}{\left(\frac{x-x^{\prime }}{l}\right)}^{2}\right) \end{array}$$

where δ is one if x and $x^{\prime }$ in the kernel function are the same input and zero otherwise.

## 4. Experiments Results and Discussion

### 4.1. CNN Training Results

**Figure 6:**
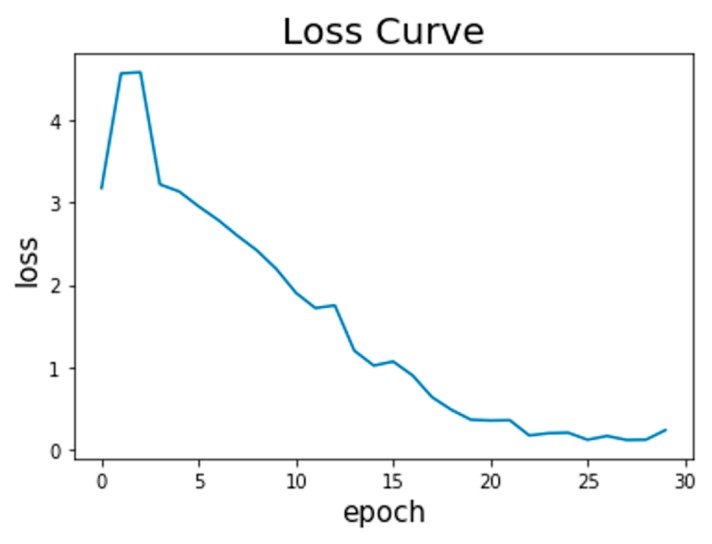
Loss curve of CNN with the 30th epoch.

Figure 6 displays an overview of the CNN training process. It is clear that the loss is below 0.3 even after the 25th epoch.

The epoch number describes the number of times that the algorithm will be trained by the entire training samples. The loss, which means the cross-entropy loss, is utilized to measure the error at the softmax activation layer. The closer the loss is to zero, the more perfect the model.

The epoch number is set to 30. It takes approximately 22.5 min totally and 45 s per epoch to finish the CNN training on a laptop equipped with 8 GB RAM and an Intel Core i5-6200 U CPU with 2.3 GHz clock.

### 4.2. GPR Training Results

For the purpose of applying a GPR model, GPy, from the Sheffield machine learning group, was utilized to design a GP framework in python. GPy 1.9.6 packet was selected to evaluate the performance of the GPR model with three different kernel functions. Prior to training the GPR model, some related parameters are presented in Section 4.3. These previous parameters were continually adjusted during the training and the final parameters will be used to describe the trained model. 

The changes in the parameters of the three kernel functions before and after training are illustrated in Table 1.

For GP, the log marginal likelihood of the model (14) is seen as the objective function of the model being optimized. The variance in Table 1 is the signal variance and is also considered as an output-scale amplitude and the length-scale in Table 1 is the input length-scale that determines the degree of correlation between inputs. Both parameters are factors that determine the smoothness of the functions estimated by a GPR. After training, variance and length-scale of both methods increase significantly. These indicate that the GPR model after training is more adaptive to the volatility of the RSSI signal and the correlation between inputs is stronger. The specific estimation process of hyperparameters is shown in Section 3.6. When the objective function of different kernel function models reaches the minimum value, each hyperparameter element in the hyperparameter vector is optimal.

Before comparing the localization accuracy of GPR models based on different kernel functions, let us take a look at the changes of the objective function values before and after the model training.

Table 2 provides a comparison of objective function values for the three kernel functions. What stands out in Table 2 is that the GPR model with Matern kernel function has the lowest objective function value after training. Meanwhile, the model with SE kernel has the second lowest value and the model with PER kernel has the highest value. What is striking about the values in Table 2 is how well the three models with different kernels fit the training data.

### 4.3. Localization Accuracy

We will demonstrate the localization cumulative error distribution (CED) of five different algorithms including CNN, CNN and GPR with SE kernel function, CNN and GPR with PER kernel function, CNN and GPR with Matern kernel function and KNN algorithm separately and compare their localization effects. The KNN algorithm is one of the algorithms with the highest localization accuracy in [29]. The literature only gives the comparison of the 75th percentile error between five algorithms among 15 months on version 1.0 database, however it does not show more detail. Therefore, we reproduced the KNN algorithm based on the version 2.1 database as a reference for precision comparison. As the setting in [29], we set k to 9.

**Figure 7:**
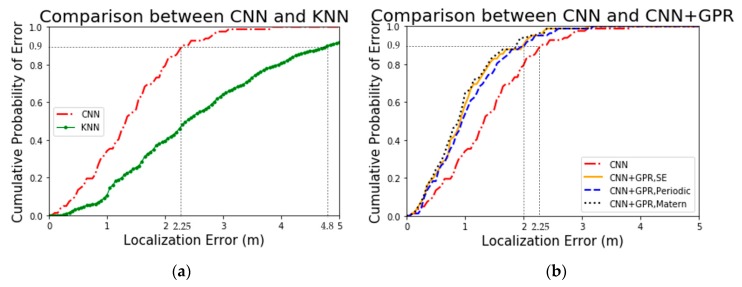
Comparisons of localization CED concerning CNN algorithm including: (**a**) Comparison between CNN and KNN; (**b**) Comparison between CNN and CNN+GPR.

From the curves demonstrated in Figure 7, it is apparent that the CNN and GPR combined model exerts significant influence on the localization accuracy. Firstly, compared to the KNN algorithm, the CNN algorithm has a significant improvement in positioning accuracy. Specifically, 90% localization error of the proposed CNN algorithm is less than 2.25 m, yet of the KNN algorithm is less than 4.80 m. Moreover, all localization error of the proposed CNN algorithm are less than 3.80 m. Secondly, the addition of the GPR model makes the localization accuracy of the improved CNN algorithm even greater. Accurately, 90% localization error of the CNN+GPR model is less than 2.00 m while of the proposed CNN algorithm is less than 2.25 m. In other words, all localization error of the CNN+GPR model is less than 3.60 m.

**Figure 8:**
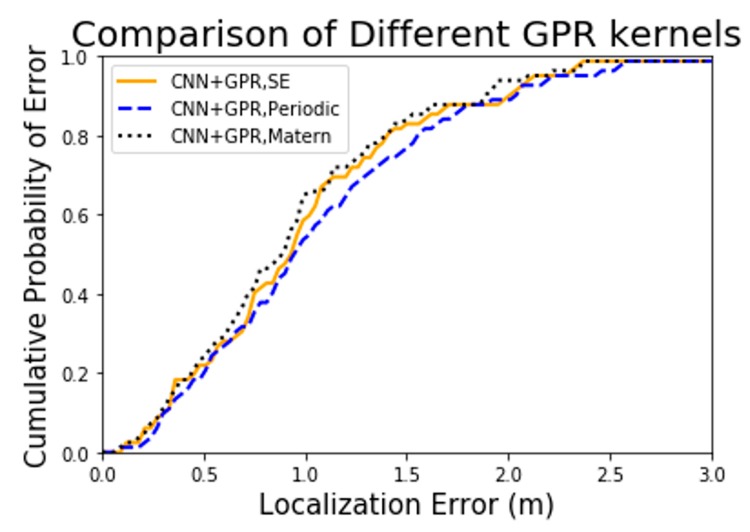
Comparison of localization CED for models with three different kernel functions.

From Figure 8, it can be seen that models with different kernel functions both have positive impacts on positioning but differ in detail. From a qualitative perspective, the model with Matern kernel function performs best in localization performance while with PER kernel function performs the worst.

**Figure 9:**
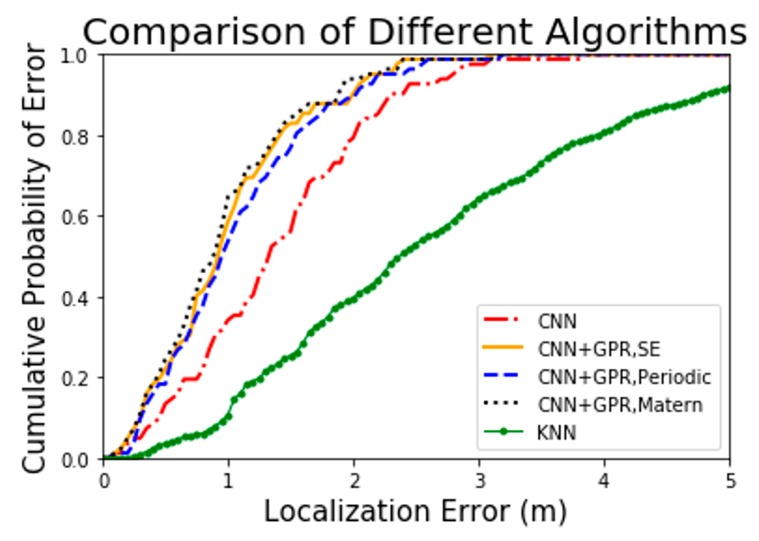
Comparison of localization cumulative error distribution (CED) for five different algorithms.

From a qualitative perspective, we can see the overall localization effects of the five algorithms from Figure 9. Particularly, the three combined algorithms, based on the CNN algorithm, employ GPR model to correct the error and achieve significant results. Meanwhile, CNN algorithm also achieves an ideal result. From a quantitative perspective, we will find more details of localization accuracy for various algorithms through mean absolute errors (MAEs) and 75th percentile errors in Table 3.

What stands out in Table 3 is the different localization accuracy for five algorithms represented by the MAEs and 75th percentile errors. The first four algorithms are the new algorithms proposed in this paper. Besides, the fifth algorithm, KNN, is employed in literature [29]. One concern expressed regarding the KNN algorithm in [29] is its evaluation that is based on version 1.0 rather than the latest version database. To be fair, we reproduced the KNN algorithm based on the version 2.1 database as the reference algorithm. Meanwhile, we exhibited the 75th percentile error of the KNN algorithm in [29].

According to Table 3, a number of issues are identified. The CNN and GPR hybrid algorithm has the best accuracy and the average localization accuracy of the three hybrid algorithms is 1.3542 m which decreases 29.4% and 61.8% error as compared with the CNN algorithm and KNN algorithm. Concentrating on the three hybrid algorithms, the algorithm with Matern kernel improves the localization accuracy by 2.0% and 12.0% compared to the algorithm with SE and PER, respectively. Additionally, the localization accuracy of the CNN algorithm is higher than the KNN algorithm by 45.8%.

Overall, the most striking finding is that both of the CNN algorithm and the hybrid algorithm based on three different kernel functions greatly improve the localization accuracy compared with the KNN algorithm which performs well in literature [29]. In addition, regarding the performance for the three kernels in this study, the results support the idea that Matern kernel function performs best in both the regression model and the localization test.

### 4.4. Supplementary Experiment

As for the practical application of the hybrid model, there are two solutions to solve real-time problems. First, the sample taken at a time can be copied into a 16 × 16 “image”. After experimental verification, this solution does not lead to a significant decrease in positioning accuracy. Second, the acquisition frequency can be increased to more than 100 Hz to provide enough samples [36]. The following will focus on the experimental verification of the first solution.

**Figure 10:**
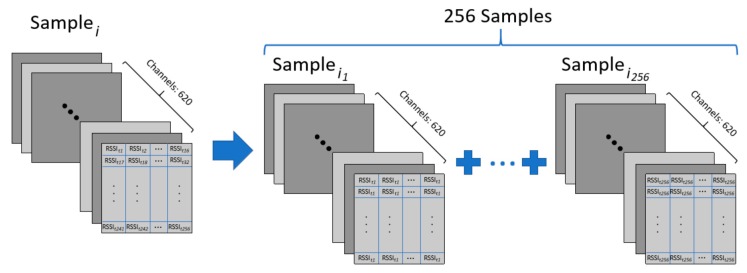
Samples conversion.

When the target object moves quickly, its dwell time at a certain point is extremely short. This may result in insufficient time to collect enough signal data and cause the model to be unavailable. We have adjusted the data preprocessing to cope with this situation. In details, the samples consisting of multiple time signals in the test dataset and validation dataset is changed to be copied from a single time signal. Since the signal at each moment can be expanded to one sample, the sample size of the test dataset and the validation dataset is greatly increased. As shown in Figure 10, each test sample is converted to 256 new test samples. The samples in the training dataset remain unchanged.

The experimental results of the solution are shown in Table 4.

Table 4 highlights the comparison of 75th percentile errors between the supplementary experiment and original experiment. From a qualitative perspective, the positioning performance exhibited by all algorithms is close to the original experiment. The hybrid algorithm is superior to CNN. The performance of the Matern kernel function is optimal in the hybrid algorithms. From a quantitative perspective, the positioning accuracy of most algorithms is very close to the original experiment. PER kernel function is not very suitable for the new test samples, resulting in a certain degree of drop in the accuracy of its hybrid algorithm.

From the data in Table 4, we can see that the localization precision of the same algorithm in the two experiments is quite close. The results indicate that the first solution proposed in the first paragraph of Section 4.4 does not lead to a significant decrease in localization precision. The above experiments prove that the proposed algorithms have certain application value.

## 5. Conclusions

This paper has proposed a wireless indoor localization model using convolutional neural network and Gaussian process regression. The most remarkable result to emerge from the data is that the CNN and GPR hybrid model improves the positioning precision by 61.8% in total compared to the baseline algorithm. While CNN has improved the performance by 45.8%, the GPR algorithm further enhanced the localization accuracy. This result has further strengthened our conviction that either the hybrid model or the CNN model adapts to complex scenes and can be treated as a reference method in complex indoor localization.

## Figures and Tables

**Table 1:** Comparison of Squared Exponential (SE) parameters before and after training.

Kernel Functions	Before/After Training	Variance	Length-Scale	Gaussian Noise	Periodic
SE	Before training	1.0	0.2	1.0	
	After training	5.0593	0.7013	0.5440 × 10^−2^	
Periodic (PER)	Before training	1.0	0.2	1.0	1.0
	After training	11.91	3.638	0.7328 × 10^−6^	0.9371
Matern	Before training	1.0	0.2	1.0	
	After training	9.966	1.619	0.2074 × 10^−4^	

The parameters after the training round four significant figures.

**Table 2:** Comparison of the objective function values for the three kernel functions.

	Before Training	After Training
SE kernel function	38.08	27.76
PER kernel function	38.22	28.69
Matern kernel function	39.45	27.34

The objective function values in Table 2 round four significant figures.

**Table 3:** The mean absolute errors (MAEs) and 75th percentile errors for different algorithms.

	Algorithm	MAE (m)	75th Percentile Error (m)	Improvement
1	CNN	1.3910	1.9188	45.8%
2	CNN+GPR (Gaussian process regression) with SE kernel	0.9989	1.3125	62.9%
3	CNN+GPR with PER kernel	1.0645	1.4625	58.7%
4	CNN+GPR with Matern kernel	0.9554	1.2875	63.7%
	Average of CNN+GPR	1.0063	1.3542	61.8%
5	k-nearest neighbor (KNN) (reference)	2.6338	3.5425	0%
	KNN ([29], version 1.0 database)		3.4060	

**Table 4:** Comparison of 75th percentile errors between supplementary experiment and original experiment.

	Algorithm	MAE (m)	75th Percentile Error (m)	Rate of Change Compared to Table 3
1	CNN	1.4420	1.8350	−4.37%
2	CNN+GPR with SE kernel	1.0073	1.3250	+0.95%
3	CNN+GPR with PER kernel	1.2218	1.6750	+14.53%
4	CNN+GPR with Matern kernel	0.9512	1.2750	+0.97%
	Average of CNN+GPR	1.0601	1.4250	+5.37%
5	KNN (reference)	2.6338	3.5425	
	KNN ([29], version 1.0 database)		3.4060	
